# Association between Ki-67 Labeling index and Histopathological Grading of Glioma in Indonesian Population

**DOI:** 10.31557/APJCP.2020.21.4.1063

**Published:** 2020-04

**Authors:** Emilia Theresia, Rusdy Ghazali Malueka, Sofia Pranacipta, Bidari Kameswari, Kusumo Dananjoyo, Ahmad Asmedi, Adiguno Suryo Wicaksono, Rahmat Andi Hartanto, Ery Kus Dwianingsih

**Affiliations:** 1 *Department of Anatomical Pathology, *; 2 *Department of Neurology, *; 4 *Division of Neurosurgery, Department of Surgery, Faculty of Medicine, Public Health, and Nursing, Universitas Gadjah Mada (FK-KMK UGM), Dr. Sardjito General Hospital, Yogyakarta, *; 3 *Department of Anatomical Pathology, Dr. Soeradji Tirtonegoro General Hospital, Klaten, Central Java, Indonesia. *

**Keywords:** Ki-67- labeling index- glioma- grading- Indonesia

## Abstract

**Background:**

Gliomas are the most frequent primary brain tumors. According to World Health Organization guidelines, gliomas are graded into four groups (Group I-IV). This histological grading will determine prognosis and treatment of the patient. Morphological criteria are not always accurate. Tumor proliferation index is a potent quantitative marker for tumor behavior and prognosis, also it’s the basis of gliomagenesis. Ki-67 immunohistochemistry examination for determining proliferation index has been suggested as an ancillary marker in deciding the definitive grading of glioma.

**Objective:**

To analyze the correlation between Ki-67 labeling index and histopathological grading of glioma in Indonesian population.

**Methods:**

One hundred and six formalin fixed-paraffin embedded tissue of glioma patients were collected from 4 different hospitals. Expression of *Ki-67* was detected using immunohistochemistry staining and the labeling index was counted. The association between Ki-67 labeling index and histopathological grading was analyzed.

**Results:**

Age range of patient were 1-73-years old, with male predominance (55.70%). Glioblastoma was the most common diagnosis accounting for 41.51% of all samples. Ki-67 labeling index cut point of 6.35% was obtained and significantly sensitive and specific for determining low- or high-grade glioma (p<0.001).

**Conclusion:**

A significant association between Ki-67 labeling index and histopathological grading in Indonesian glioma patients has been revealed. The result of this study may be used to improve diagnostic and grading accuracy of glioma cases in Indonesia, especially in small biopsy specimens.

## Introduction

Data from The Global Cancer Atlas showed that 5.323 people i n Indonesia were diagnosed with primary brain and central nervous system cancer in 2018. Gliomas are the most frequent among all primary brain tumors (Rasmussen et al., 2017; Bray et al., 2018). Glioma histological grading classification based on World Health Organization (WHO) was divided into 4 grades (grade I-IV). This grading determines the prognosis and treatment for the patient but there are still high inter- and intraobserver variability (Nielsen et al., 2018). 

The basic of gliomagenesis was the glial cell proliferation. Tumor proliferation index is a potent quantitative marker for tumor behavior and prognosis. Therefore, it has been suggested as an ancillary marker in deciding the definitive grading of glioma. It can be examined with a few immunohistochemistry markers. Among them, Ki-67 has been established as an independent factor for tumor progression and survival in glioma patients (Darweesh et al., 2016; Byreddy et al., 2018). 

Skjulsvik et al., (2014) reported that high-grade gliomas (WHO grade III/IV) have significantly higher Ki-67 labeling index than low-grade gliomas (WHO grade I/II). Ki-67 labeling index 10% has been used as cut-off point for differentiating low-grade and high-grade glioma because of its significances for predicting patient’s survival. However, due to no precise guidelines for Ki-67 labeling index evaluation, there were overlapping values between histological grades. Also, the reported Ki-67 labeling index values varied between studies (Nielsen et al., 2018).

This study is trying to establish standardized and reproducible approaches in evaluation of Ki-67 labeling index in glioma and analyzing the association between Ki-67 labeling index and histopathological grading of glioma in Indonesian population. This report is expected to reinforce the application of Ki-67 immunostaining as an ancillary tool in determining the accurate grading of glioma for appropriate management. 

## Materials and Methods

*Samples and data collection*

We retrospectively identified, by reviewing databases from four referral hospitals in Yogyakarta region, all patients between 1 January 2010 and 31 July 2019 with a confirmed diagnosis of glioma. Eligible patients were required to have their formalin-fixed paraffin-embedded (FFPE) tumor tissue blocks available. In total, 106 samples were collected. All pathological specimens were reviewed and reclassified according to WHO Classification of Tumors of the Central Nervous System 2016 by expert pathologists. Tumors were graded based on morphological criteria. Glial fibrillary acid protein immunostaining and IDH-1 mutation status examination were performed in all tumor tissue blocks. The study was approved by the Medical and Health Research Ethics Committee of the Faculty of Medicine, Public Health, and Nursing, Universitas Gadjah Mada, Yogyakarta, Indonesia.

*Immunohistochemistry staining*

FFPE samples were cut into 3µm thick slides for immunostaining examination. FFPE sections were incubated, deparaffinized, and rehydrated. Antigen retrieval was performed using a decloaking chamber (BioCare Medical, USA). Mouse monoclonal antibody Ki-67/MIB 1 (BioCare Medical, USA) was diluted to 1:100 in phosphate buffer saline. Diamino-benzidine then applied for visualization of positive cells, continued with Hematoxylin staining as counterstain. Tonsil tissue was used as positive control for Ki-67 staining. 

*Interpretation of Ki-67 immunostaining results*

Ki-67 positive cells were evaluated by an independent and experienced pathologist under a light microscope in high power field (HPF). Areas that will be evaluated were photographed. Evaluation of Ki-67 labeling index was using average method by 2 independent pathologists without knowing the tumor grading and results of the other pathologist’s Ki-67 interpretations. 

This photomicrograph method of Ki-67 was adapted from Leung et al., (2016). As much as 1,000 tumor cells were counted, maximum number of cells counted in 1 photomicrograph were 500 cells. Every tumor nucleus that stained brown in any intensity was considered positive. Number of positive nuclei were divided by total number of counted nuclei and the values were presented as percentage. The average values between 2 pathologists were calculated for the final Ki-67 labeling index. 

*Statistical analysis*

The association between Ki-67 labeling index and glioma grading were analyzed using Kruskal-Wallis and Chi-square test. A probability value of less than 0.05 was considered as statistically significant.

**Figure 1:**
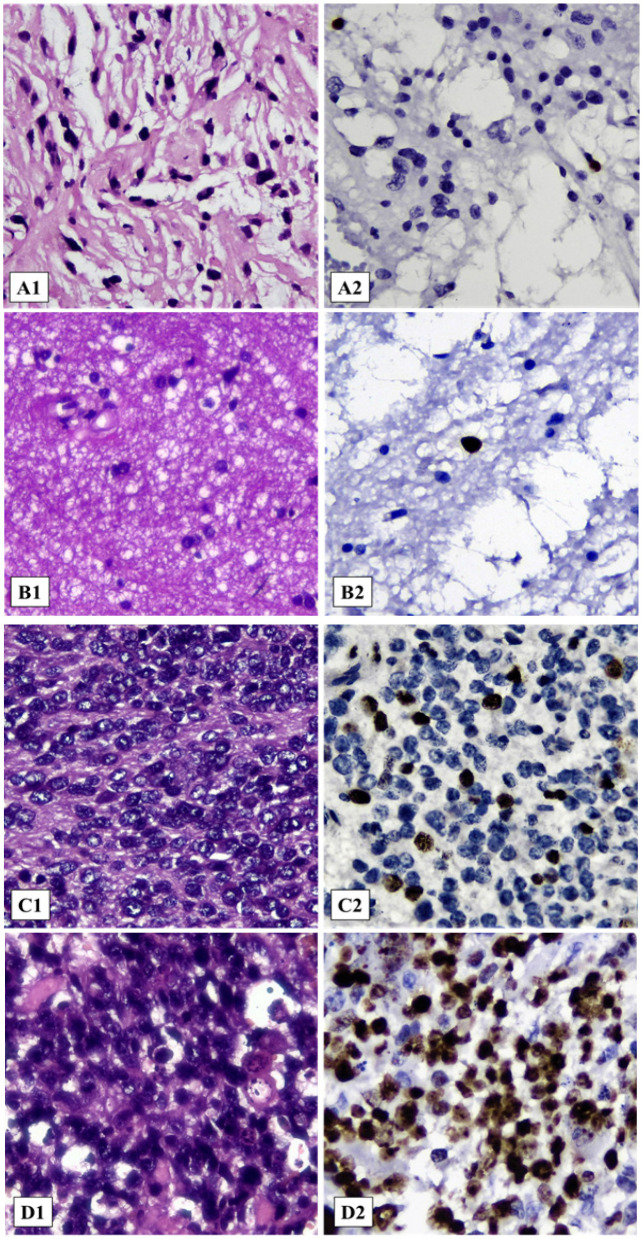
Histopathological Features of each Grade of Glioma and Its Ki-67 Labeling Index. (A) Glioma WHO grade I, sample PG-04, diagnosed as Pilocytic astrocytoma, IDH-wildtype. (A1) Microscopic appearance (HE, 400x). (A2) Immunostaining of Ki-67 with Ki-67 labeling index = 1.26% (400x). (B) Glioma WHO grade II, sample FG-66, diagnosed as Diffuse astrocytoma, IDH-wildtype. (B1) Microscopic appearance (HE, 400x). (B2) Immunostaining of Ki-67 with Ki-67 labeling index = 4.90% (400x). (C) Glioma WHO grade III, sample FG-52, diagnosed as Anaplastic oligodendroglioma, IDH-wildtype. (C1) Microscopic appearance (HE, 400x). (C2) Immunostaining of Ki-67 with Ki-67 labeling index = 16.45% (400x). (D) Glioma WHO grade IV, sample FG-71, diagnosed as Glioblastoma, IDH-mutant. (D1) Microscopic appearance (HE, 400x). (D2) Immunostaining of Ki-67 with Ki-67 labeling index = 73.55% (400x).

**Table 1:** Demographic and Clinicopathological Features of the Subjects

Characteristics		Frequency	Percentage
Gender			
Male		59	55.70%
Female		47	44.30%
Age			
Children (0-17 years old)		22	20.76%
Adult (18-64 years old)		78	73.58%
Elderly (≥65 years old)		6	5.66%
Range of age	1-73		
Mean of age	38.47		
WHO grade and histopathological diagnosis			
I			
Pylocytic Astrocytoma, IDH-wildtype		6	5.66%
Pylocytic Astrocytoma, IDH-mutant		1	0.94%
II			
Diffuse astrocytoma, IDH-wildtype		16	15.11%
Diffuse astrocytoma, IDH-mutant		7	6.60%
Gemistocytic astrocytoma, IDH-wildtype		1	0.94%
Gemistocytic astrocytoma, IDH-mutant		1	0.94%
Oligodendroglioma, IDH-mutant		3	2.83%
Oligoastrocytoma, IDH-mutant		3	2.83%
Ependymoma		4	3.77%
Clear Cell Ependymoma		1	0.94%
III			
Anaplastic Astrocytoma, IDH-wildtype		8	7.55%
Anaplastic Oligoastrocytoma, IDH-wildtype		2	1.89%
Anaplastic Oligodendroglioma, IDH-wildtype		4	3.77%
Anaplastic Oligodendroglioma, IDH- mutant		3	2.83%
Anaplastic Ependymoma		2	1.89%
IV			
Glioblastoma, IDH-wildtype		33	31.13%
Glioblastoma, IDH-mutant		8	7.55%
Giant Cell Glioblastoma, IDH-wildtype		2	1.89%
Gliosarcoma, IDH-wildtype		1	0.94%

**Table 2:** Distribution of Ki-67 Labeling Index in each Grade of Glioma

		Ki-67 labeling index (%)					
		Mean	Standard Deviation	Median	Minimum	Maximum	p*
Grade	Grade I	1.24	1.23	0.90	0.15	3.78	<0.001
	Grade II	3.96	3.75	3.25	0.34	18.15	
	Grade III	23.02	13.51	23.00	1.90	55.75	
	Grade IV	23.88	16.41	20.90	0.85	73.55	

*Kruskall-Wallis test, p value <0.05 considered as statistically significant

**Table 3:** The Correlation between Ki-67 Labeling index and Glioma Grading

		Grade						
		Low		High		p*	OR**	CI 95%***
		Count	%	Count	%			
Ki-67 labeling index	≤6.35	40	88.9%	5	11.1%	<0.001	18.07	5.96-54.74
	>6.35	3	4.9%	58	95.1%			

*Chi-square test analysis, p value <0.05 considered as statistically significant; ** OR, Odds ratio; ***CI95%, Confident interval of 95%

**Figure 2:**
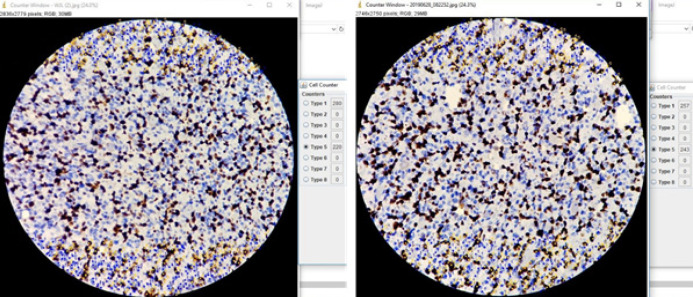
Average Method of Ki-67 Labeling Index Adapted from Leung et al., (2016). Counting immunopositively cells (yellow dot) from 1000 tumor cells (immunonegative cells = blue dot) using ImageJ program. Sample FG-38, WHO grade IV, diagnosed as Glioblastoma, IDH-mutant with Ki-67 labeling index results = 44.95%.

**Figure 3:**
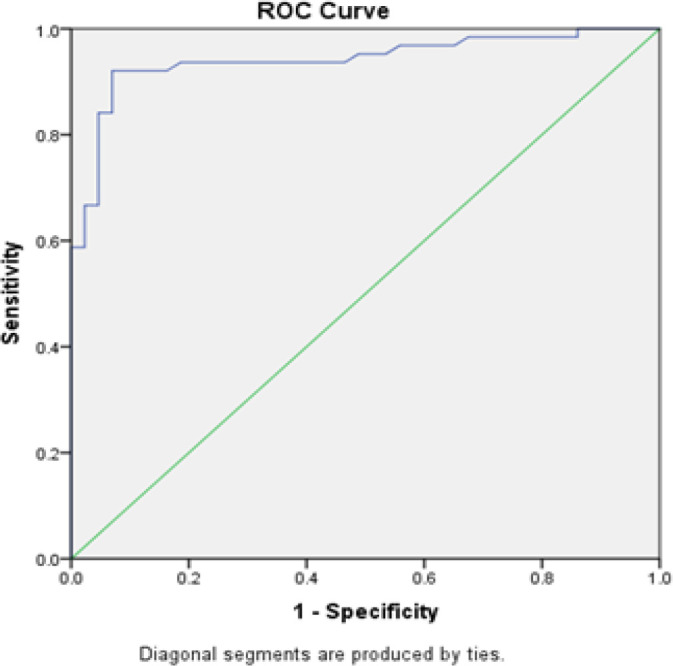
Graphs Showed ROC Curve for Ki-67 Labelling Index. From the ROC curve, using the Youden index method, optimal cut-off point in Ki-67 labeling index of 6.35% was obtained with 92% sensitivity and 93% specificity

## Results

*Demographic and Clinicopathological features of glioma subjects*

The demographic and clinicopathological features of the research subjects are summarized in Table 1. Age range of all subjects was 1 to 73 years old (mean 38.47 years old). Majority of the patients were adult patients (73.58%) and male (55.70%). From the histopathological examination according to the WHO 2016 classification, there were 7 (6.60%) samples of grade I, 36 (33.96%) samples of grade II, 19 (17.93%) samples of grade III, and 44 (41.51%) samples of grade IV. The most common histopathological diagnosis was Glioblastoma, IDH-wildtype.

*Immunohistochemistry Analysis of Ki-67*

Histopathological features of each grade of glioma and its Ki-67 labeling index in the tissue samples are shown in Figure 1. The nuclear immunoreactivity of the Ki-67 in any degree was considered as positive and further used for analysis. 

*Ki-67 Labeling Index and Glioma Grading*

The lowest Ki-67 labeling index was 0.15% and the highest was 73.55%. Distribution of Ki-67 labeling index for each grade of glioma was shown in Table 2. Analysis using Kruskall-Wallis test showed significant difference of Ki-67 labeling index among the four grades (p<0.001). To elaborate more about this, analysis of Ki-67 labeling index using receiver operating characteristic (ROC) curve was performed and optimal cut-off value at 6.35% was obtained with sensitivity of 92% and specificity of 93% (Figure 3). By using this cut off value a significant association was found between Ki67 labelling index and grade of glioma (p<0.001) (Table 3). 

## Discussion

Glioma is the most frequent primary brain tumor, especially glioblastoma. It is more prevalent in males compared to females. Incidence of high-grade glioma tends to increase with age (Louis et al., 2016; Rasmussen et al., 2017; Ostrom et al., 2018). This was also described in our study with male patient predominance (male: female = 1.26: 1), the youngest mean age was in glioma grade I (14.71 years old) while the highest mean age was in grade IV (46.50 years old) and the most common diagnosis was Glioblastoma, IDH-wildtype, (31.13%). 

There are 4 histological gradings in glioma. The determination of the grade is a subject for interobserver variability. Most of the time, the distinction between grade II and III especially in diffuse glioma based solely on mitotic activity (Olar et al., 2015; Louis et al., 2016). Glioma then was separated into 2 large groups, low-grade glioma (grade I and II) and high-grade glioma (grade III and IV) with distinct differences in terms of prognosis and therapy of choice (Hsu et al., 2003). 

Skjulsvik (2014) dan Byreddy (2018) stated that there was a significant increase in Ki-67 labeling index in high-grade glioma compared to low-grade glioma but there were overlapping values between grade III and IV. This is similar to our study, where the mean value of Ki-67 labeling index of grade III (23.02%) was very similar to grade IV (23.88%). Therefore, the Ki-67 labeling index has limitation in differentiating glioma per grade but useful in differentiating between low- and high-grade glioma.

Establishing a Ki-67 cut-off value is also a topic of interest in many studies. This value should be able to distinguish between low-grade glioma and high-grade glioma. The most common cut-off value of Ki-67 labeling index used for glioma was 10%. Several studies showed that this value was clinically significant and associated with the survival of the patients (Johannessen and Torp, 2006; Tavares et al., 2016). However, until now there is no standardized procedure for immunostaining and counting of Ki-67, causing great inter- and intra-observer variability. In this case, each pathology department should establish its Ki-67 labeling index cut-off value accompanied by regular adjustment with tumor grading and survival rate for laboratory quality assurance (Skjulsvik et al., 2014). In our study the cut-off value was 6.35% which was similar to Byreddy et al., (2018) which showed a cut-off value of 6%. This cut off value had high sensitivity and specificity in differentiating low-grade and high-grade glioma (92 % and 93% respectively). This is higher compared to previous studies which showed 77% sensitivity and 75% specificity in discrimination of grade II and III astrocytomas (Sallinen et al., 1994). Commonly used Ki67 cut off value of 10% was also statistically significant in determining low- or high-grade glioma in our patients. However, this cut off value resulted in a wider range of confident interval and lower sensitivity (data not shown).

Glioma grading based only on morphology can be inaccurate especially in small biopsy specimens, usually due to heterogeneity-induced sampling errors. In cases where clinical and radiological parameters have lack of correlation with histopathological results and tumor grade, Ki-67 labeling index can be used as an ancillary test (Thotakura et al., 2014). High proliferation rate in histological benign lesion may indicate there was a higher-grade tumor or a reactive condition. To differentiate between reactive lesion and true neoplasm, analyses for isocitrate dehydrogenase and p53 mutation combined with clinical and radiological assessment can be helpful (Kurtkaya-Yapıcıer et al., 2002; Capper et al., 2010). If there is any suspicion for high-grade glioma, we should add the information of elevated Ki-67 labeling index in the diagnosis with the recommendation of close clinical follow-up. On the contrary, high-grade glioma with low proliferation should not be down-graded (Trembath et al., 2008). 

Other factors also can contribute to the discrepancy between Ki-67 labeling index and glioma gradings such as preanalytical factors (e.g. tissue fixation, tissue processing, paraffin block storage) and immunostaining procedure (e.g. antibody selection, antigen retrieval process). But the most important factor is the quality of immunohistochemistry interpretation by the pathologist (Gown, 2016). 

There are no precise guidelines for estimating Ki-67 labeling index in gliomas, therefore intra- and inter-observer variability, also overlapping values between histological grades have been reported. Several counting methods to evaluate Ki-67 labeling index for gliomas have been proposed. Most common method for Ki-67 in gliomas is the manual hot-spot method but because gliomas can have tumor heterogeneity there is no certainty that the most intense hot spot exists in the biopsy section. Assessment of the whole slide with average method is more recommendable due to its reproducibility and accuracy but required a semi-automatic quantitative approach (Hsu et al., 2003; Jang et al., 2017; Nielsen et al., 2018). Study from Leung et al. (2016) was trying to established a standardized scoring protocol for Ki-67 using calibration test (Leung et al., 2016). We adapted the protocol for counting Ki-67 from the study. Using this protocol combined with average counting method, we reach high interobserver agreement (Kappa index = 0.96). The Ki-67 counting results can be seen in Figure 2. 

This study has several strong points. One of them is the larger number of samples compared to previous studies. We also used the newest glioma classification according to the WHO Classification of Tumours of the Central Nervous System 2016 edition that used integration of histological grading and molecular parameters. The Ki-67 labeling index interpretation used average counting method that covered the heterogeneity nature of glioma. A more standardized scoring protocol was also used, which is more accurate and easily reproducible. This method also decreased the intra- and inter-observer variability, which was the main problem in Ki-67 labeling index studies. The weakness of this study is the distribution of glioma per grade was not equal, which may influence the results.

In conclusions, in this study, a significant association between Ki-67 labeling index and histopathological grading in Indonesian glioma patients has been revealed. The Ki-67 labeling index is useful in differentiating between low- and high-grade glioma together with histopathological morphology, clinical and radiological parameters. The result of this study may be used to improve diagnostic and grading accuracy of glioma cases in Indonesia, especially in small biopsy specimens.
